# The Expression of the Nectin Complex in Human Breast Cancer and the Role of Nectin-3 in the Control of Tight Junctions during Metastasis

**DOI:** 10.1371/journal.pone.0082696

**Published:** 2013-12-26

**Authors:** Tracey A. Martin, Jane Lane, Gregory M. Harrison, Wen G. Jiang

**Affiliations:** Cardiff University-Peking University Cancer Research Institute, Cardiff School of Medicine, Cardiff University, Heath Park, Cardiff, United Kingdom; Wayne State University School of Medicine, United States of America

## Abstract

**Introduction:**

Nectins are a family of integral protein molecules involved in the formation of functioning Adherens and Tight Junctions (TJ). Aberrant expression is associated with cancer progression but little is known how this effects changes in cell behaviour. This study aimed to ascertain the distribution of Nectins-1 to -4 in human breast cancer and the effect on junctional integrity of Nectin-3 modulation in human endothelial and breast cancer cells.

**Methods:**

A human breast tissue cohort was processed for Q-PCR and immunohistochemistry for analysis of Nectin-1/-2/-3/-4. Nectin-3 over-expression was induced in the human breast cancer cell line MDA-MB-231 and the human endothelial cell line HECV. Functional testing was carried out to ascertain changes in cell behaviour.

**Results:**

Q-PCR revealed a distinct reduction in node positive tumours and in patients with poor outcome. There was increased expression of Nectin-1/-2 in patients with metastatic disease, Nectin-3/-4 was reduced. IHC revealed that Nectin-3 expression showed clear changes in distribution between normal and cancerous cells. Nectin-3 over-expression in MDA-MB-231 cells showed reduced invasion and migration even when treated with HGF. Changes in barrier function resulted in MDAN3 cells showing less change in resistance after 2h treatment with HGF (p<0.001). Nectin-3 transformed endothelial cells were significantly more adhesive, irrespective of treatment with HGF (p<0.05) and had reduced growth. Barrier function revealed that transformed HECV cells had significantly tighter junctions that wildtype cells when treated with HGF (p<0.0001). HGF-induced changes in permeability were also reduced. Overexpression of Nectin-3 produced endothelial cells with significantly reduced ability to form tubules (p<0.0001). Immunoprecipitation studies discovered hitherto novel associations for Nectin-3. Moreover, HGF appeared to exert an effect on Nectin-3 via tyrosine and threonine phosphorylation.

**Conclusions:**

Nectin-3 may be a key component in the formation of cell junctions and be a putative suppressor molecule to the invasion of breast cancer cells.

## Introduction

The Nectins are a family of immunoglobulin-like cell adhesion molecules that have long been thought of as essential components for the formation of cell–cell adhesions and regulators of cellular functions that include cell polarization, differentiation, movement, proliferation and survival [1]. The Nectin family is comprised of four members, Nectin-1 (PVRL1 (Poliovirus receptor-related 1), HveC (herpesvirus entry mediator C), CD111 (Cluster of Differentiation 111)), Nectin-2 (PVRL2 (Poliovirus receptor-related 2), HveB (herpesvirus entry mediator B), CD112 (Cluster of Differentiation 112), Nectin-3 (PVRL3 (Poliovirus receptor-related 3), CD113) and Nectin-4. The four members of the Nectin family are thought to be ubiquitously expressed and have a number of spliced variants.

Each Nectin has a c-terminal motif of 4 amino acids (E/A-K-Y-V) that interacts with the PDZ domain of afadin. Nectin-1 has two splicing variants, nectin-1α and –1β/HigR [2]–[3]. Nectin-2 also has two splicing variants, nectin-2α and –2δ [4]–[5]. Nectin-3 has three splicing variants nectin-3α, -3β and –3δ [6]. The extracellular regions of splicing variants are identical, but their transmembrane regions and cytoplasmic regions are different. The cytoplasmic regions of nectin-1α, -2α, -2δ, -3α and 3δ have a C-terminal conserved motif of 4 amino acid residues (E/A-X-Y-V), which interact with the PDZ domain of afadin through which they are liked to the actin cytoskeleton [7]. The physiological role of Nectins has yet to be satisfactorily clarified [8], although work suggests that they may play a key role in the proper organisation of both adherens junctions (AJ) and tight junctions (TJ) [9].

These Ca (2+)-independent cell adhesion molecules first form cell-cell adhesions where cadherins are recruited, forming adherens junctions in epithelial cells and fibroblasts. In addition, Nectins recruit claudins, occludin, and junctional adhesion molecules (JAM's) to the apical side of AJs, forming TJs in epithelial cells. All four Nectin family members have one extracellular region with three Ig-like loops, one transmembrane segment and one cytoplasmic tail [10]. The formation of cis-dimers is necessary for the formation of Nectin trans-dimers. Nectin-3 was first described by Satoh-Horikawa [6] as a member of the Nectin family. The first Ig-like loop of Nectin-3 is essential and sufficient for the formation of trans-dimers with Nectin-1, but the second Ig-like loop of Nectin-3 was furthermore necessary for its cell-cell adhesion activity [10].

Although Nectins were initially thought to be only localised at AJs, studies have suggested that a role in the formation or organisation of TJs may be found. Reymond et al. [11] showed that Nectin-3 (PRR3) interacts with afadin by interaction of the C-terminal to the PDZ domain of afadin. Inagaki et al. [12] have shown that the Nectin-afadin system is able to recruit ZO-1 to the Nectin-based cell-cell adhesion sites in non-epithelial cells that have no TJs.

Besides their role in physiology, Nectins have been involved in different pathological processes in humans where they serve as virus receptors (poliovirus and herpes simplex virus), they are involved in orofacial malformation (CLPED1) and recently they have been described as markers, actors and potential therapeutic targets in cancer [13]–[14]. Nectin-2 and Nectin-4 are often overexpressed in tumours, and are associated with a poor prognosis [14]. Indeed, Nectin-2 has been found to be over-expressed in clinical breast and ovarian cancer tissues by using gene expression profile analysis and immunohistochemistry studies [15]. Nectin-2 was over-expressed in various cancer cell lines as well [13]. Interestingly, a polyclonal antibody specific to Nectin-2 suppressed the in vitro proliferation of OV-90 ovarian cancer cells, which express endogenous Nectin-2 on the cell surface. The anti-Nectin-2 antibpdies generated were classified into 7 epitope bins. The anti-Nectin-2 mAbs demonstrated antibody-dependent cellular cytotoxicity (ADCC) and epitope bin-dependent features such as the inhibition of Nectin-2-Nectin-2 interaction, Nectin-2-Nectin-3 interaction and *in vitro* cancer cell proliferation. A representative anti-Nectin-2 mAb in epitope bin VII, Y-443, showed anti-tumour effects against OV-90 cells and MDA-MB-231 breast cancer cells in mouse therapeutic models, and its main mechanism of action appeared to be ADCC. These findings suggest that Nectin-2 is a potential target for antibody therapy against breast and ovarian cancers [15].

The expression of Nectin-4 was increased in ovarian cancer compared with normal ovaries. Reverse transcriptase-polymerase chain reaction (RT-PCR) and quantitative RT-PCR validated the overexpression of Nectin-4 messenger RNA in ovarian cancer compared with normal ovarian cell lines and tissues. Protein levels were elevated in ovarian cancer cell lines and tissue compared with normal ovarian cell lines. Cleaved Nectin-4 was detectable in a number of patient serum samples and in patients with benign gynecologic diseases with high serum CA125 levels, Nectin-4 was not detected in the majority of cases, suggesting that it may serve as a potential biomarker that helps discriminate benign gynecologic diseases from ovarian cancer in a panel with CA125. Fabre-Lafay et al. [16] also found Nectin-4 not to be detected in normal breast epithelium. By contrast, Nectin-4 was expressed in 61% of ductal breast carcinoma vs 6% in lobular type. Expression of Nectin-4 strongly correlated with the basal-like markers EGFR, P53, and P-cadherin, and negatively correlated with the luminal-like markers ER, PR and GATA3. All but one ER/PR-negative tumour expressed Nectin-4. The detection of Nectin-4 in serum improved the follow-up of patients with MBC as the association of CEA/CA15.3/Nectin-4 allowed monitoring of 74% of these patients compared to 67% with the association CEA/CA15.3 [16]. Serum Nectin-4 was also found to be a marker of therapeutic efficiency and correlates, in 90% of cases, with clinical evolution. The authors concluded that Nectin-4 was a new tumour-associated antigen for breast carcinoma and a new bio-marker whose use could help refine breast cancer taxonomy and improve patient follow-up [16].

In lung cancer, Maniwa et al. [17] demonstrated that of 127 patients, 25% showed membranous expression of Nectin-3, and others showed negative or cytoplasmic expression. Membranous expression of Nectin-3 was found to be a prognostic factor for decreased overall survival and Multivariate Cox proportional hazards model analyses revealed that membranous expression of Nectin-3 was an independent prognostic factor. In tumours expressing membranous Nectin-3, some did not co-localize with E-cadherin and these patients showed poorer prognosis than other patients for overall survival. Conversely, membranous expression of Nectin-3 with E-cadherin co-localization was found to associate with good prognosis of patients.

Breast cancer is the most common cancer in women worldwide and is the principle cause of death from cancer among women globally. Despite the high incidence rates, in Western countries, 89% of women diagnosed with breast cancer are still alive 5 years after their diagnosis, which is due to detection and treatment [18]. The UK and USA have one of the highest incidence rates worldwide (together with the rest of North America and Australia/New Zealand), making these countries a priority for breast cancer awareness (Worldwide Breast Cancer statistics). Breast cancer has been the most common cancer in the UK since 1997, despite the fact that it is rare in men (Cancer Research UK Statistics). It is by far the most common cancer among women in the UK (2010), accounting for 31% of all new cases of cancer in females. In 2010, there were 49,961 new cases of breast cancer in the UK with 49,564 (99%) in women and 397 (less than 1%) in men, giving a female to male ratio of around 125 to 1. Despite rising survival rates, mainly due to earlier detection and better treatments, many of the cellular processes underlying the disease remain to be determined. Aberrant expression of Nectins has been associated with cancer and evidence has shown that Nectins may be integral to the correct functioning of TJs. TJs in epithelial cells act as cell-cell adhesion structures and govern paracellular permeability. Disruption of these functions often leads to the dissociation and metastasis of cancer cells.

There has not been, to date, a study examining the distribution and expression of all four Nectins in human breast cancer and further information on the roles of Nectin-3 have yet to be determined. This study aimed to ascertain the distribution of Nectins in human breast cancer and to determine the role that Nectin-3 may have in regulating cell behaviour in human breast cancer and endothelial cells.

## Materials and Methods

### Ethics statement

All research involving human tissues was under the ethical approval of the local ethics committee (Bro Taf Local Research Ethics Committee (Panel B) for the Bro Taf Health Board, Cardiff, UK, issued 10/12/2001, reference 01/4303). All data was analysed anonymously and informed verbal consent given. As the tissues were collected before the introduction of the Human Tissue Act, UK, 2004, no written consent was necessary and documentary measures not required. The project license (PPL 30/2591) under which all *in vivo* work was carried out was approved by both the Cardiff University School of Medicine JBIOS Committee and the UK Home Office under the Animals (Scientific Procedures) Act 1986, as issued by the Secretary of State for the UK Home Office. In vivo work was carried out under the strict guidelines of the UK Home Office to ensure that the 3R's were strictly adhered to. Thus, the minimum number of animals was used in the experiment, with the minimum of suffering and maximum attention to animal welfare. The maximum severity band allowed was moderate, although the procedures carried out in this work were ostensibly only mild. Animals were checked daily and their behavior and health monitored. Animals were weighed and measured twice weekly to ascertain loss of health (as determined by weight loss greater than 20% or tumour burden greater than 1cm3). Adverse effects resulted in sacrifice via UK Schedule One procedures.

### Reagents and antibodies

Nectin-1, -2 and -3 (A is C-19; B is K-20) antibodies were obtained from Santa-Cruz Biotechnologies Inc. (Santa Cruz, USA) as was anti-actin (sc-8432), anti-Occludin, ZO-1, ZO-2, ZO-3, AF6, Ezrin, Moesin, Radixin, E-cadherin, SIPA-1, α-catenin, β-catenin, γ-catenin, Axin, MAGI-1, MAGI-2, MAGI-3, CAR, Tricellulin and ROCKI. Secondary peroxidase conjugated antibodies were from Sigma (Sigma-Aldrich, Dorset, UK).

### Cell lines and culture conditions

The human breast cancer cell lines MDA-MB-231 and MCF-7 were obtained from ECACC and the HECV endothelial cell line from the Interlab Cell Line Collection (ICLI), Naples, Italy and were routinely maintained in Dulbecco's Modified Eagle Medium/F12 (DMEM/F12) (Sigma-Aldrich, Dorset, UK) supplemented with 10% fetal calf serum (FCS), penicillin and streptomycin (Sigma-Aldrich, Dorset, UK). The cells were incubated at 37°C, 5% CO2 and 95% humidity.

### Human breast specimens

The human breast cancer tissue cohort consisted of a total of 133 breast samples obtained from breast cancer patients (106 breast cancer tissues and 27 associated background or related normal tissue), with the consent of the patients and local ethical committee approval (Bro Taf Healthboard). The tissues were verified by a pathologist as normal background and cancer specimens, and it was confirmed that background samples were free from tumour deposit. The tissues were immediately frozen in liquid nitrogen following excision.

### RNA extraction and Reverse Transcription-Polymerase Chain Reaction (RT-PCR)

Cells were grown to confluence in a 25 cm^3^ flask before RNA was extracted using total RNA isolation (TRI) reagent and following the protocol provided (Sigma-Aldrich, Dorset, UK). RNA was converted to cDNA using iScript cDNA synthesis kit (Primer Design Ltd., Southampton, UK). Following cDNA synthesis, samples were probed using actin primers to check the quality of the cDNA and confirm uniform levels within each sample together with those specific for the transcript (full primer sequences are outline in Table 1). Conventional PCR was performed using a T-Cy Thermocycler (Beacon Technologies Ltd., The Netherlands) using REDTaq® ReadyMix™ PCR Reaction mix (Sigma-Aldrich, Dorset, UK). Cycling conditions were as follows: 94°C for 5 min, 94°C for 30 s, 55°C for 30 s, 72°C for 30 s and the final extension phase at 72°C for 7 min for 36 cycles. The PCR products were separated on a 2% agarose gel and electrophoretically separated. The gel was then stained with ethidium bromide prior to examine under ultraviolet light and photographs taken. The primers used are shown in Table 1.

**Table 1:** Primers for PCR used in this study.

Primer name	Sequence (5′-3′)
β-actin	βactinF- atgatatcgccgcgctcg; βactinR- cgctcggtgaggatcttca
GAPDH	GAPDHF1- aaggtcatccatgacaactt; GAPDHR1- ggctgcttttaactgggta
Nectin-1	NectinF1- attacgggaaaaagacacag
Nectin-2	Nectin2F1- tgatacctgtgaccgtctct; Nectin2R1- aggatgaaggccaaggac
Nectin-3	Nectin3F1- agttatggagggtgacttga; Nectin3R1- atggctgctactgtttcatt
Nectin-4	Nectin4F1- aaggatcacccacatcct; Nectin4R1- acataggccacctgctt
Nectin3FX1*	Nectin3FX1- gcaaagcacaactttcctc
Nectin3FX2*	Nectin3FX2- ggaaaatacatctgcaaagc
Nectin3FX3*	Nectin3FX3- atgaaacagtagcagccatt
Nectin3FX4*	Nectin3FX4- atggcgcggaccctgcggc
Nectin3FX5*	Nectin3FX5-cttcattttgtccatccatt
Nectin3FX6*	Nectin3FX6- tggctggaatattctgctat
Nectin3RX	Nectin3RX- ctaaacataccactccctcct
Nectin-1Z**	NectinF1- attacgggaaaaagacacag; Nectin1ZR- acgaacctgaccgtacaagttcttggtcaccctttct
Nectin-2Z**	Nectin2F1- tgatacctgtgaccgtctct; Nectin2ZR- actgaacctgaccgtacaagctcactcggattatatgc
Nectin-3Z**	Nectin3F- agttatggagggtgacttga; Nectin3ZR- actgaacctgaccgtacacattctttccccatactgt
Nectin-4Z**	Nectin4F1- aaggatcacccacatcct; Nectin4ZR- actgaacctgaccgtacacacttgagcatagctccttc
GADPHZ**	GAPDH2F- ctgagtacgtcgtggagtc; GAPDHZR2- actgaacctgaccgtacacagagatgatgacccttttg
β-actinZ**	βactinZF- ggacctgactgactacctca; βactinZR- actgaacctgaccgtacaagcttctcctt

Nectin3FX1 to FX6 versus Nectin3RX; **Z primers for Q-PCR.

### Real-time quantitative Polymerase Chain Reaction (Q-PCR)

The Amplifluor system was used to detect and quantify transcript copy number of Nectin-1, Nectin-2 and Nectin-3 in tumour and background samples. Primers were designed by Beacon Designer software, which included a complementary sequence to universal Z probe (Intergen, Inc.). Each reaction contains 10 pmol reverse primer (which has the Z sequence), 10 pmol of FAM-tagged universal Z probe (Intergen, Inc.) and cDNA (equivalent to 50 ng RNA) (primer sequences are shown in Table 1). Sample cDNA was amplified and quantified over a large number of shorter cycles using an iCyclerIQ thermal cycler and detection software (BioRad laboratories, Hammelhempstead, UK) under the following conditions: an initial 5 minute 94°C period followed by 60 cycles of 94°C for 10 seconds, 55°C for 15 seconds and 72°C for 20 seconds. Detection of GAPDH copy number within these samples was later used to allow further standardisation and normalisation of the samples. Q-PCR primers are shown in Table 1.

### Over-expression of Nectin-3 in MDA-MB-231 breast cancer cells and HECV endothelial cells

A range of normal human tissues were screened for Nectin-3. Normal breast tissue was chosen for endogenous expression of Nectin-3. The human breast cancer cell line MDA-MB-231 and the human endothelial cell line were chosen for introduction of the Nectin-3. The gene, after amplification from breast tissue cDNA was cloned into aPEF6/V5-His TOPO TA plasmid vector (Invitrogen Ltd., Paisley, UK) before electroporation into the cells. Expression of the gene was confirmed by RT-PCR. The Nectin-3 expression construct and empty plasmid were, respectively, used to transfect MDA-MB-231 and HECV cells by electroporation. Stably transfected cells were then used for subsequent assays after being tested at both transcriptional and translational level. Those cells containing the expression plasmid and displaying enhanced Nectin-3 expression were designated MDAN3 and HECVN3, those containing the closed pEF6 empty plasmid and used as control cells were designated MDApEF6 and HECVpEF6 and unaltered wild type were designated MDAWT and HECVWT. Expression primers were: Nectin3EXF1: 5′-atggcgcggaccctgcggccgtc-3′ and Nectin3RX 5′-ctaaacataccactccctcct-3′.

### Construction of hammerhead ribozyme transgene targeting human Nectin-3

Hammerhead ribozymes that specifically target human Nectin-3 were constructed based on its secondary structure. Touch down PCR was used to generate PCR-based ribozymes which were then cloned into a pEF6/V5-His vector (selection markers: ampicillin and blasticidin, for prokaryotic and mammalian cells respectively), and amplified in *Escherichia coli*, purified, verified and used for electroporation into both MDA-MB-231 and MCF-7 human breast cancer cells lines. The targets were as follows: Nectin3ribR1-actagtacaatgcctgtcaaaacttttcgtcctcacggact and Nectin3rib1F-ctgcagaacggtgagatatgccttgctgatgagtccgtgagga.

### SD-PAGE, Western blotting and co-immunoprecipitation

Cells were grow to confluence, detached and lysed in HCMF buffer containing 0.5% SDS, 0.5% Triton X-100, 2 Mm CaCl2, 100 µg/ml phenylmethylsulfonyl fluoride, 1 mg/ml leupeptin, 1 mg/ml aprotinin and 10 Mm sodium orthovanadate for 1 hour, sample buffer was added and the protein boiled at 100°C for 5 min before being spun at 13,000 g for 10 min to remove insolubles. Protein concentration was quantified using Bio-Rad Protein Assay kit (Bio-Rad Laboratories, Hertfordshire, UK). Equal amounts of protein from each cell sample were added onto a 10% or 15% (depending on protein size) acrylamide gel and subjected to electrophoretic separation. The proteins were transferred onto nitrocellulose membranes which were blocked and probed with specific primary antibodies (1∶500), following with peroxidase-conjugated secondary antibody (1∶1000). Protein bands were visualized with Supersignal West Dura system (Perbio Science UK Ltd., Cramlington, UK) and detected using a CCD-UVIprochemin system (UVItec Ltd., Cambridge, UK). Co-immunoprecipitation samples were prepared as follows: cell lysate of the protein of interest was probed with primary antibodies (1∶100 dilution) and placed on a rotating wheel for 2 hour allowing primary antibodies to bind to their targets. One hundred microlitres of conjugated A/G protein agarose beads (Santa-Cruz Biotechnologies Inc., USA) were added to each sample to make the antibody-protein complex insoluble, followed by overnight incubation on the rotation wheel. The supernatant was discarded and the pellet was washed in 200 µl of lysis buffer and resuspended in 200 µl of 2X Lamelli sample buffer concentrate (Sigma-Aldrich, Dorset, UK), then denatured for 5 minutes by boiling at 100°C.

### Trans-epithelial resistance (TER) and Paracellular Permeability

Cells were seeded into 0.4 µm transparent pore size inserts (Greiner bio-one, Stonehouse, UK) at a density of 50,000 cells in 200 µl of medium within 24 well plates, grown to confluence, the medium removed and replace with fresh Dulbecco's Modified Eagle's medium containing 15 Mm Hepes, L-Glutamine (Lonza Laboratories, Verviers, Belgium). Medium alone was added to the base of the wells (control) or with 40 or 50 ng/ml HGF [19]. Resistance across the layer of cells was measured using an EVON volt-ohmmeter (EVOM, World Precision Instruments, Aston, Herts, UK), equipped with static electrodes (WPI, FL, USA) for a period of 4 h. Paracellular permeability (PCP) was determined using fluorescently labeled dextran FITC-Dextran 40, molecular weight being 40 kDa. Human breast cancer cells were prepared and treated as in the TER study, but with the addition of Dextran-40 to the upper chamber. Medium from the lower chamber was collected for intervals up to 2 h after addition of HGF. The relative fluorescence from these collections was read on a multichannel fluorescence reader (Denly, Sussex, UK).

### *In vitro* cell growth assay

MDA-MB-231 and HECV cells were seeded into a 96 well plate at a density of 3,000 cells/well to obtain density readings after 1 day, 2 days, 3 days, 4 days and 5 days. Within each experiment four duplicates were set up. After appropriate incubation periods, cells were fixed in 4% formaldehyde in BSS for 5–10 minutes before staining for 10 minutes with 0.5% (w/v) crystal violet in distilled water. The crystal violet was then extracted from the cells using 10% acetic acid. Absorbance was determined at a wavelength of 540 nm on a plate reading spectrophotometer.

### *In vitro* cell matrix adhesion assay

The cell-matrix attachment was carried out as previously described method [19]. Briefly, 45,000 cells were seeded onto the Matrigel basement (10 µg/well) membrane in 200 µl of normal medium and incubated at 37°C with 5% CO2 for 40 minutes. After the incubation period, the medium was aspirated and the membrane washed 5 times with 150 µl of BSS to remove the non-attached cells, then fixed in 4% formaldehyde (v/v) in BSS for 10 minutes before being stained in 0.5% crystal violet (w/v) in distilled water. The number of adherent cells were counted from 5 random fields per well and 5 duplicate wells per sample, under a microscope.

### *In vitro* invasion cell assay

Cell culture inserts (BD Falcom™ Cell Culture Inserts, BD Bioscience, Erembodegem, Belgium) were placed into a 24-well plate using forceps and coated in Matrigel (BD Biosciences, Oxford, UK). The working solution of Matrigel was prepared at a concentration of 0.5 mg/ml, adding 100 µl to each insert and allowed to dry overnight. Once dried the inserts were rehydrated in 100 µl sterile water for 1 hour. The water was then aspirated and cells were seeded in the inserts over the top of the artificial basement membrane at a density of 30,000 cells in 200 µl per well. The plates were then incubated for 3 days at 37°C at 5% CO2. After the incubation period, the Matrigel layer together with the non-invasive cells was cleaned from the inside of the insert with a tissue paper. The cells which had migrated through the pores and invaded into the Matrigel, were fixed in 4% formaldehyde (v/v) in BSS for 10 minutes before being stained in 0.5% crystal violet (w/v) in distilled water. The cells were then visualized under the microscope under ×40 magnification, 5 random fields counted and duplicate inserts used for each test sample.

### *In vitro* Cytodex-2-bead motility assay

Cells were pre-coated onto Cytodex-2 beads (GE Healthcare, Cardiff, UK) for 2 hours. The medium was aspirated and the beads washed in medium to remove non-adherent or dead cells. The beads were resuspended in 5 ml of medium. Cell were aliquoted into a 24-well plate, 5 duplicate wells per sample (300 µl/well), and incubated overnight. Following incubation, cells that had migrated from the Cytodex-2 beads and adhered to the base of the wells were washed gently in BSS, fixed in 4% formaldehyde (v/v) in BSS for 10 minutes before being stained in 0.5% crystal violet (w/v) in distilled water. Five random fields per well were counted under the microscope.

### Tubule formation assay

A volume of 100 µl serum free medium containing 250 µg Matrigel (250 µg/well) was seeded in a 96 well plate and left to gel in an incubator for 30 minutes, followed by a heating oven until dry. Before use, the Matrigel was rehydrated in 100 µl of serum free medium and cells seeded at a density of 40,000 cells/well. Following incubation at 37°C for 1 hour, the medium was aspirated and a second layer of Matrigel was added followed by incubation at 37°C 3 for 30 minutes to gel. Medium was then added and the cells left overnight to allow tubules to form.

### *In vivo* development of mammary tumours

Athymic nude mice (nu/nu) were purchased from Charles River Laboratories (Charles River Laboratories, Kent, UK) and maintained in filter top units according to Home Office regulations and ethical requirements. Each group of mice consisted of 5 mice and each mouse was injected with a mix of 2×10^6^ cancer cells in 100 µl in a 0.5 mg/ml Matrigel suspension in both flanks. Two groups were included: MDA-MB-231^pEF6^ and MDA-MB-231^N3exp^. The mice were weighed and tumour size measured twice weekly using a vernier calipers under sterile conditions. Those mice that developed tumours exceeding 1 cm^3^ or suffered 25% weight loss during the experiment were terminated under Schedule 1 according to the UK Home Office and the UK Coordinating Committee on Cancer Research (UKCCCR) instructions. Tumour volume was determined using the following formula: tumour volume = 0.523×width2×length.

### Statistical analysis

Statistical analysis was performed by MINITAB version 13.32 (Minitab Inc. State College, PA, USA) using a two-sample student t-test and the non-parametric Mann-Whitney confidence interval test or Kruskal-Wallis, where appropriate.

## Results

### Expression of Nectins in human breast cancer at the transcript level

**Figure 1:**
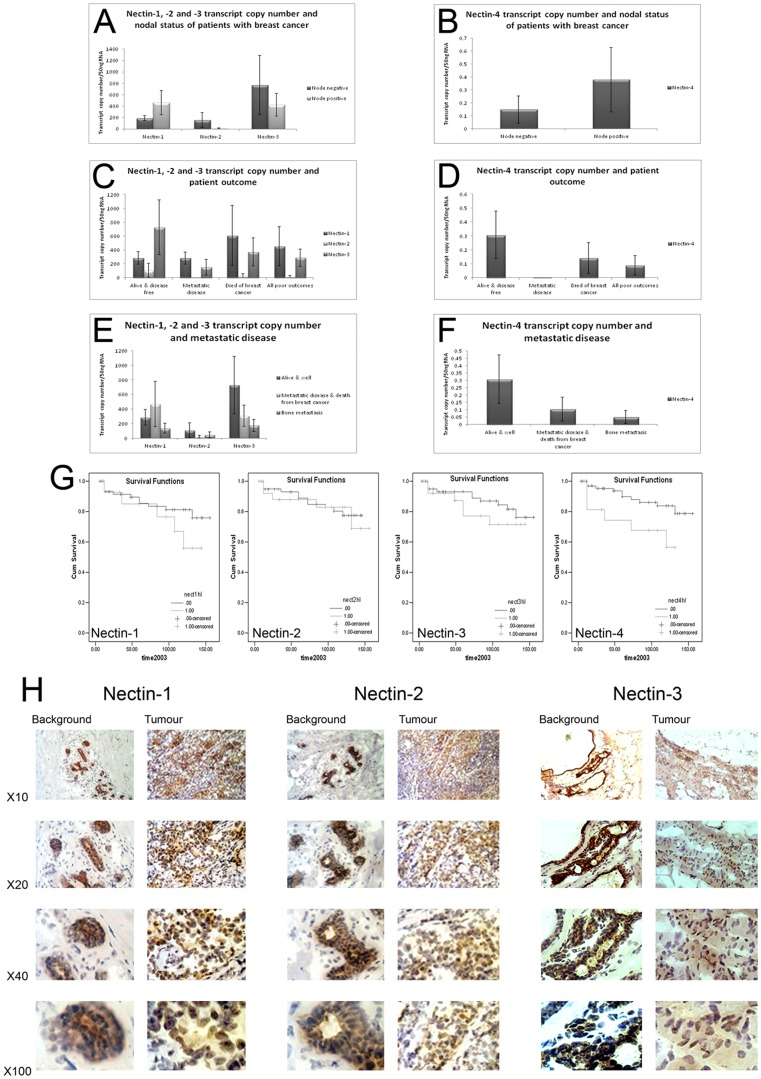
Expression of the Nectin family in human breast cancer. (A) and (B) Expression of Nectin-1 to -4 and nodal status of breast cancer patients at the transcript level. (C) and (D) Transcript levels and patient outcome. (E) and (F) Nectin transcript expression and metastatic disease in human breast cancer. (G) Nectins and patient survival. (H) Immunohistochemistry of Nectin-1 (left), Nectin-2 (middle) and Nectin-3 of representative patients tissues.

Patient tissue was prepared for both mRNA and protein analysis. Q-PCR revealed that all four Nectins had elevated levels in tumour samples, although significance was not reached (Nectin-1: tumour 304+/−96 versus background 213+/−103); Nectin-2: tumour 89.4+/−72.1 versus background 1.820+/−0.727; Nectin-3: tumour 1507+/−953, background 250+/−117; Nectin-4: tumour 0.242+/−0.122 versus background 0.134+/−0.106). However, nodal status showed that whilst Nectin-1 and -4 were increased in node positive tumours (Nectin-1 node negative 189+/−42, node positive 466+/−214; Nectin-4 node negative 0.149+/−0.10, node positive 0.38+/−0.25), both Nectin-2 and -3 were reduced (Nectin-2 node negative 154+/−130, node positive 11.1+/−9.1; Nectin-3 node negative 2458+/−1761, node positive 425+/−198), Figure 1A and B.

Nectin-1 was found to have higher expression with increasing NPI (Nottingham Prognostic Indicator), tumour grade and TNM status (tumour-nodal involvement) as was Nectin-4 (Nectin-1: NPI1 188.7+/−42 versus NPI3 560+/−500; Grade1 191.3+/−78.5 versus Grade3 1313+/−1051; TNM1 266+/−45 versus TNM3 1313+/−1051, Nectin-4: NPI1 0.149+/−0.104 versus NPI3 0.323+/−0.201; Grade1 0.0265+/−0.024 versus Grade3 0.252+/−0.2; TNM1 0.00001+/−0.000 versus TNM3 0.252+/−0.24). Expression of NEctin-2 was reduced with increasing NPI (NPI1 154+/−130 versus NPI3 32.7+/−31.3) and Grade (Grade1 29+/−26.8 versus Grade3 4.85+/−1.93) but was increased with TNM status (TNM1 0.0154+/−0.013 versus TNM3 4.85+/−1.93). Nectin-3 was reduced with increasing NPI (NPI1 2458+/−1761 versus NPI3 301+/−216) and Grade (Grade1 1701+/−1517 versus Grade3 687+/−408) and was also increased in increasing TNM status (TNM1 500.5+/−92.5 versus TNM3 687+/−408).

### Nectin expression and patient survival

When patient outcome was analysed, it was seen that Nectin-1 was elevated in patients that had died from breast cancer, and all poor outcomes overall (Figure 1C). In comparison, Nectin-2, -3 and -4 were all reduced in patients with metastatic disease and those who had died of the disease (Figure 1C and 1D). When looking more closely at those with metastatic disease, we found that this was also true for metastasis to bone (Figure 1 E and 1F), however, these did not reach significance.

Long-term survival curves were calculated using Kaplan-Meier survival curves (Fig. 1G). Patients with higher levels of Nectin-1 had shorter survival than patients with low levels (not significant); high mean survival 114.98 months (91.83–138.12 months, 95% CI) versus low mean survival 133.66 months (121.52–145.81 months, 95% CI, cut-offs as previously determined (Martin et al. 2004). This was true of Nectin-2 (high mean survival 132.46 months (113.10–151.22 months, 95% CI) versus low mean survival 127.86 months (117.64–138.07 months, 95% CI) and Nectin-3 (high mean survival 137.88 months (127.20–148.55 months, 95% CI) versus low mean survival 117.74 months (99.20–136.29 months, 95% CI). For Nectin-4, higher levels were significantly associated with better survival (high mean survival 138.82 months (128.83–148.81 months, 95% CI) versus low mean survival 97.58 months (73.24–121.93 months, 95% CI, p = 0.0274).

### Nectin expression and estrogen receptor status

Levels of Nectin-1 were elevated in estrogen receptor positive (ER+) tumours, when compared to estrogen negative (ER−) (ER+ 609+/−280 versus ER− 152.5+/−29.7). However, estrogen receptor β positive (ERβ+) had lower levels of Nectin-1 than estrogen receptor β negative (ERβ−) (ERβ+ 165.6+/−73.7 versus ERβ− 344+/−121). For Nectin-2, ER− levels were higher than ER+, whilst ERβ+ were lower than ERβ− (ER+ 1.655+/−0.513 versus ER− 149+/−121; ERβ+ 24+/−21.9 versus ERβ− 114.8+/−97.1). In Nectin-3, ER+ levels were higher than ER−, and ERβ+ were lower than ERβ− (ER+ 3693+/−3052 versus ER− 655+/−450; ERβ+ 259+/−101 versus ERβ− 1964+/−12080). This was also true for Nectin-4 (ER+ 0.404+/−0.359 versus ER− 0.144+/−0.092; ERβ+ 0.008+/−0.005 versus ERβ− 0.278+/−0.092), however, these did not reach significance.

### Nectin expression in ductal carcinomas

Expression of Nectin-1 and Nectin-2 was higher in ductal carcinomas than in all other histological types (Nectin-1: ductal 348+/−122 versus other 112+/−27, p = 0.063; Nectin-2: ductal 104+/−93 versus other 82+/−38, p = 0.0037). Ductal carcinomas had lower levels of Nectin-3 but not Nectin-4, (Nectin-3: ductal 1534+/−1177 versus other 2581+/−1699, p = 0.6; Nectin-4: ductal 0.30+/−0.15 versus other 0.022+/−0.021, p = 0.074). When considering patient outcome in those with ductal cancer, it was found that Nectin-1 was increased in those with poor outcome (with metastasis and death), but reduced with those who had bone metastasis (alive & well 315+/−135; poor outcome 554+/−360; bone metastasis 158.2+/−94.5, significance not reached). For Nectin-2, patients with ductal carcinoma who remained alive and well had higher levels than those with poor outcomes (alive & well 131+/−130; poor outcome 1.357+/−0.627; bone metastasis 1.46+/−0.48, significance not reached). Patients with ductal cancer, who remained alive and well, had higher levels of Nectin-3 than those who had poor outcome or bone metastasis (alive & well 390+/−151; poor outcome 324+/−154; bone metastasis 165+/−73.4, significance not reached). This was also the case for Nectin-4 (alive & well 0.382+/−0.207; poor outcome 0.092+−0.087; bone metastasis 0.0053+/−0.005, p = 0.07, significance not reached).

### Protein expression and distribution of Nectin-1, -2 and -3 in patient tissues

**Figure 2:**
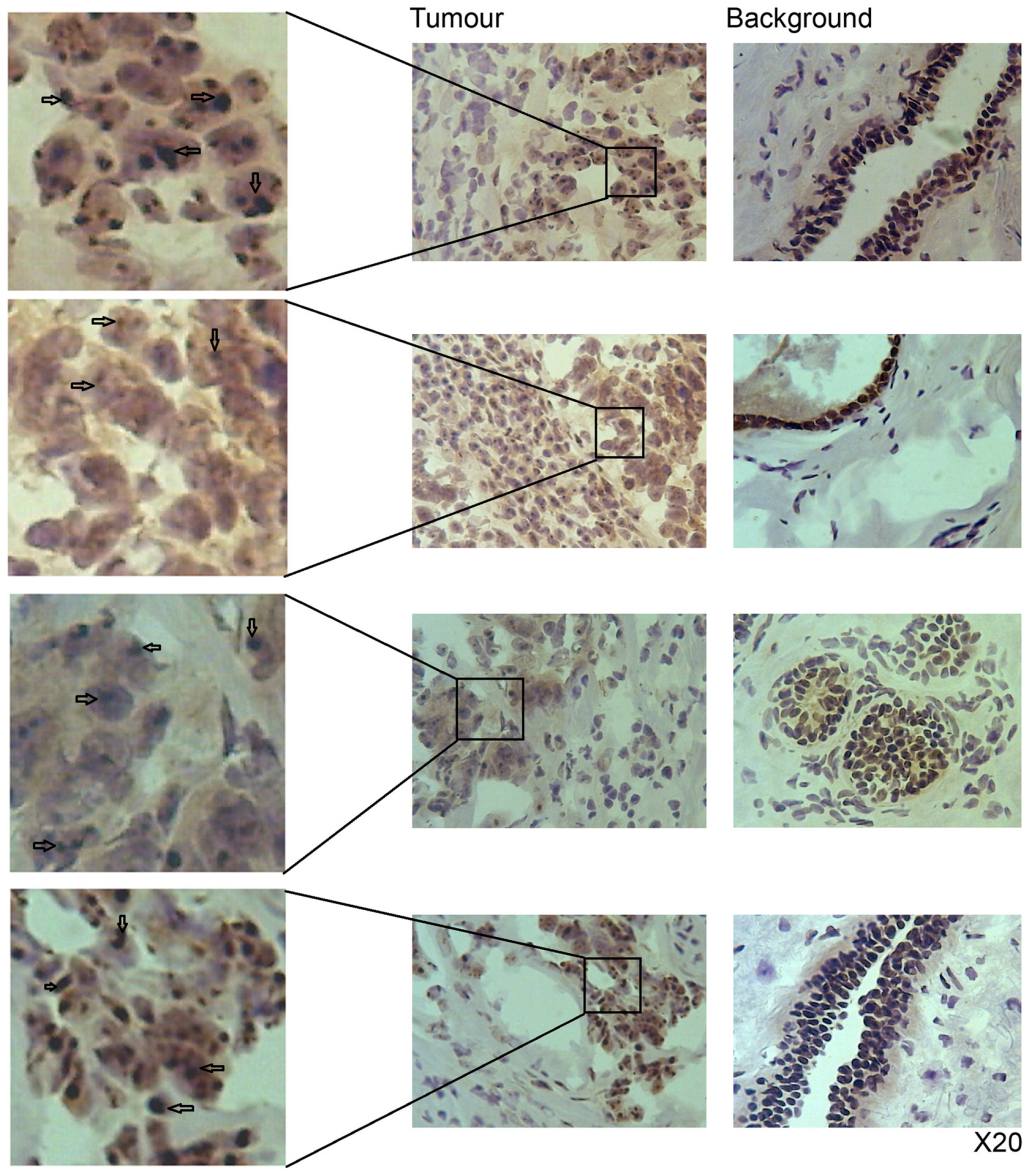
Immunohistochemical staining of Nectin-3 in human breast cancer and background tissue to show concentrated Nectin-3 inclusions in tumour sections.

When paired tumour/background tissues were screened for Nectin protein expression, there appeared little overall difference in tumour expression for both Nectin-1 and Nectin-2 (Figure 1H left and center panels), although both strongly stained tumour cells, whereas only endothelial cells were strongly stained in background tissue. However, Nectin-3 was much reduced in tumour tissues, when compared to background (Figure 1H, right panel). Further investigation showed that tumour cells had reduced staining of the cytoplasm but concentrated complexes of Nectin-3 within the nuclear area (Figure 2 left panel). This was not the case in cells in background tissues. Due to the unusual distribution we decided to investigate Nectin-3 further.

### Screening of Nectins in human cancer cell lines

**Figure 3:**
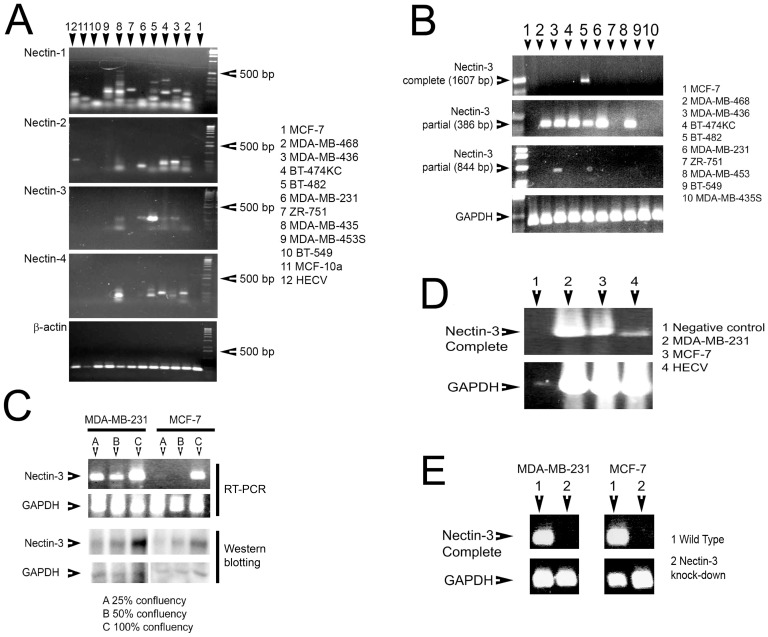
Evaluation of Nectin expression in human breast cancer cell lines using RT-PCR (A). Nectin-3 expression in human breast cancer cell lines showing sequential RT-PCR (B). Effect of cell confluency and Nectin-3 transcript and protein expression as assessed using Western Blotting (C). Confirmation of over-expression of Nectin-3 in human breast cancer and endothelial (HECV) cell lines (D).

**Figure 4:**
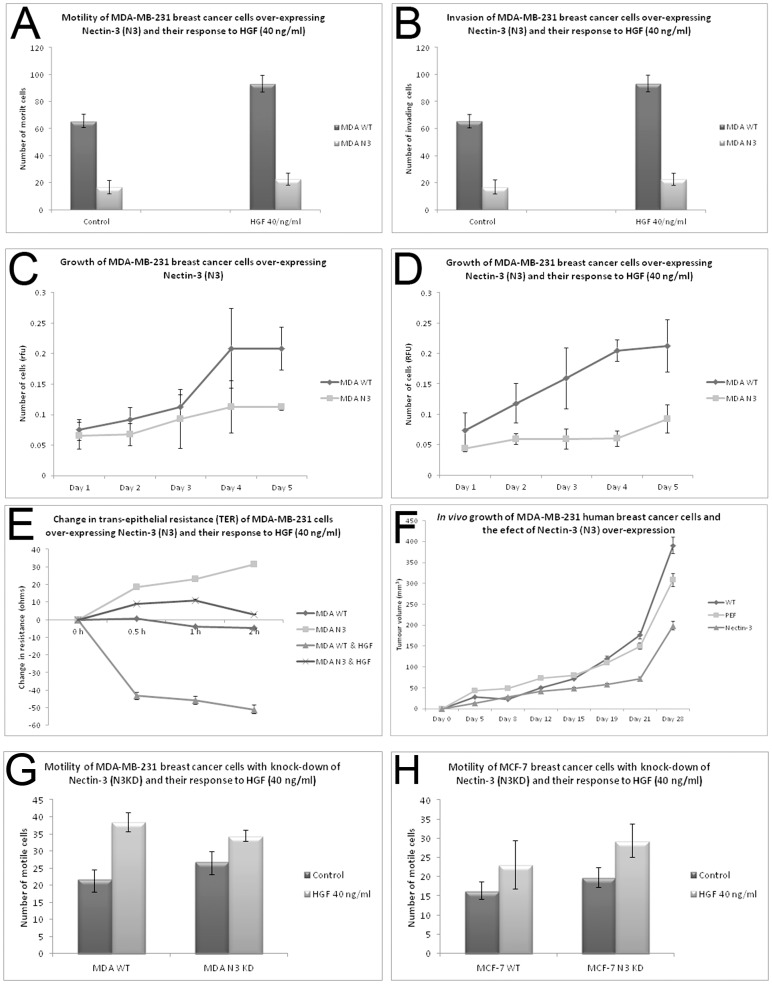
Effect of Nectin-3 over-expression and knockdown in the human breast cancer cell line MDA-MB-231. (A) Nectin-3 caused reduced motility, (B) decreased invasion, (C) and (D) reduced *in vitro* growth (even after treatment with HGF at 40 ng/ml), (E) increased trans-epithelial resistance (TER) and (F) reduced *in vivo* tumour growth. Knockdown of Nectin-3 resulted in increased motility in MDA-MB-231 (G) and MCF-7 (H) cells.

PCR primers were initially designed to amplify a 300 bp region of each Nectin for fast screening of human breast cancer and endothelial (HECV) cell lines (Figure 3A, Table 1). However, from the 12 cell lines analysed, there was no correct sized band for Nectin-1, only MDA-MB-436 and BT-474KC breast cancer cell lines expressed the correct sized band for Nectin-2, only BT-482 breast cancer cell lines strongly showed the correct band for Nectin-3 (with very weak bands for MDA-MB-436 and MDA-MB-231 cells) and Nectin-4 was not correctly expressed in any cell lines. As we wished to investigate Nectin-3 further, we designed a number of PCR primer to amplify (a) the full length of the Nectin-3 coding gene, (b) sequentially larger regions in order to ascertain where the change in Nectin-3 length of transcript arose (Table 1). Only BT-482 cells contained the complete length Nectin-3 transcript Figure 4A, top). Of all the regions amplified, only the regions amplified to produce a 386 bp and an 844 bp fragment produced the correct products (Figure 3B). All the breast cancer cells analysed had the correct fragment 386 bp fragment, apart from MCF-7, ZR-751, BT-549 and MDA-MB-435S cells. Only MDA-MB-436 cells had the 844 bp fragment. It appears from these results that Nectin-3 is expressed as a truncated form in nearly all the human breast cancer cells analysed. Although all PCR experiments were consistent and carried out a minimum of three times, we decided to examine the expression of Nectin-3 as regards to confluency. We chose two breast cancer cells lines: MDA-MB-231, aggressive cells that only expressed the 386 bp region and MCF-7 cells, less aggressive and not expressing any regions of Nectin-3. We extracted mRNA from both cell lines after growth to reach 25%, 59% and 100% confluency. We were interested to see that after using the PCR primers amplifying the 386 bp region, that transcript signal increased with increasing confluency for both cell lines (top, Figure 3C). Moreover, this was confirmed using Western Blotting (bottom, Figure 3C). We then decided to over-express Nectin-3 in MDA-MB-231 and MCF-7cells to observe any changes in cell aggressiveness.

### Construction of over expression of Nectin-3

**Figure 5:**
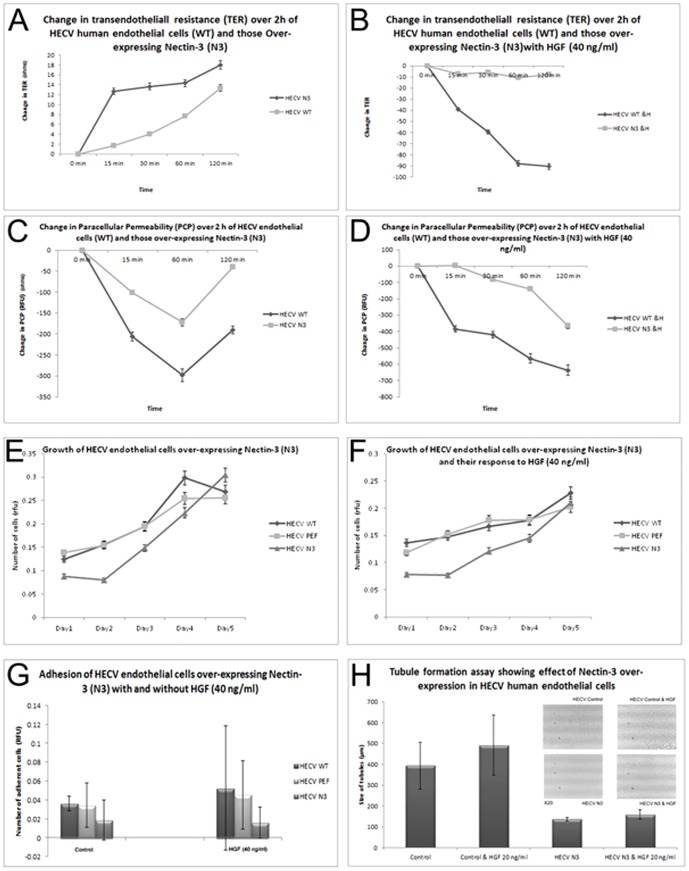
Effect of Nectin-3 over-expression in the human endothelial cell line, HECV. (A) and (B) Nectin-3 caused increased resistance and (C) and (D) reduced paracellular permeability. No change was seen in *in vitro* growth assays (E and F). Nectin-3 over-expression caused reduced adhesion (G), but increased tubule formation (H).

Nectin-3 (complete gene) was cloned into MDA-MB-231 and MCF-7 human breast cancer cells and expression confirmed using RT-PCR (Figure 5). Moreover, the human endothelial cell line, HECV also received the Nectin-3 gene for further investigation (Figure 3D).

### Effect of Nectin-3 overexpression and knock down on breast cancer cell behavior

Nectin-3 over-expression effected numerous changes on cellular behavior of the aggressive MDA-MB-231 cells. Motility was significantly reduced in modified cells (MDAN3) when compared to MDA-MB-231 wild-type cells (MDAWT), even under the influence of HGF (hepatocyte growth factor), a well-known motogen and metastasis factor (p<0.001) Figure 4A. Invasion was also significantly reduced in MDAN3 cells (p<0.02) Figure 4B. *In vitro* growth assays showed that MDAN3 cells were also significantly slower growing than MDAWT cells (Figure 4C) even under the influence of HGF (Figure 4D), p<0.05). When looking at changes in TJ function, it was found that barrier function (as measured using TER) was significantly increased in MDAN3 cells, implying that the modified cells had “tighter” TJ. This increased barrier function was also able to resist the effect of HGF, when compared to MDAWT cells (Figure 4E), p<0.001. *In vivo* tumour growth assays showed that Nectin-3 over-expression was able to significantly reduced tumour growth over 28 days (Figure 4F), p<0.05. Knock down of Nectin-3 in MDA-MB-231 cells and MCF-7 cells resulted in significantly increased motile behavior (Figure 4G), although there was little additive effect with the addition of HGF (p<0.005).

### Effect of Nectin-3 expression on human endothelial cell behavior

Over-expression of Nectin-3 effected significant changes on human endothelial cells. TER was significantly increased in HECVN3 cells, in comparison to HECVWT (Figure 5A) p, even under the influence of HGF (Figure 5B) p<0.00001. Paracellular permeability of HECVN3 cells was reduced compared to HECVWT, both in treated and non-treated cells (Figure 5C and D, p<0.05). There was however, no significant difference in growth rate of Nectin-3 over-expressed cells (Figure 5E and 5F). Adhesion of HECVN3 cells was significantly reduced compared to HECVWT/HECVPEF control cells with or without HGF (Figure 5G), p<005. When a tubule formation assay was carried out, HECVN3 cells produced tubules with significantly reduced size, even when treated with HGF, which is a strong angiogenic factor (Figure 5H), p<0.0001.

### Investigation of Nectin-3 protein binding

**Figure 6:**
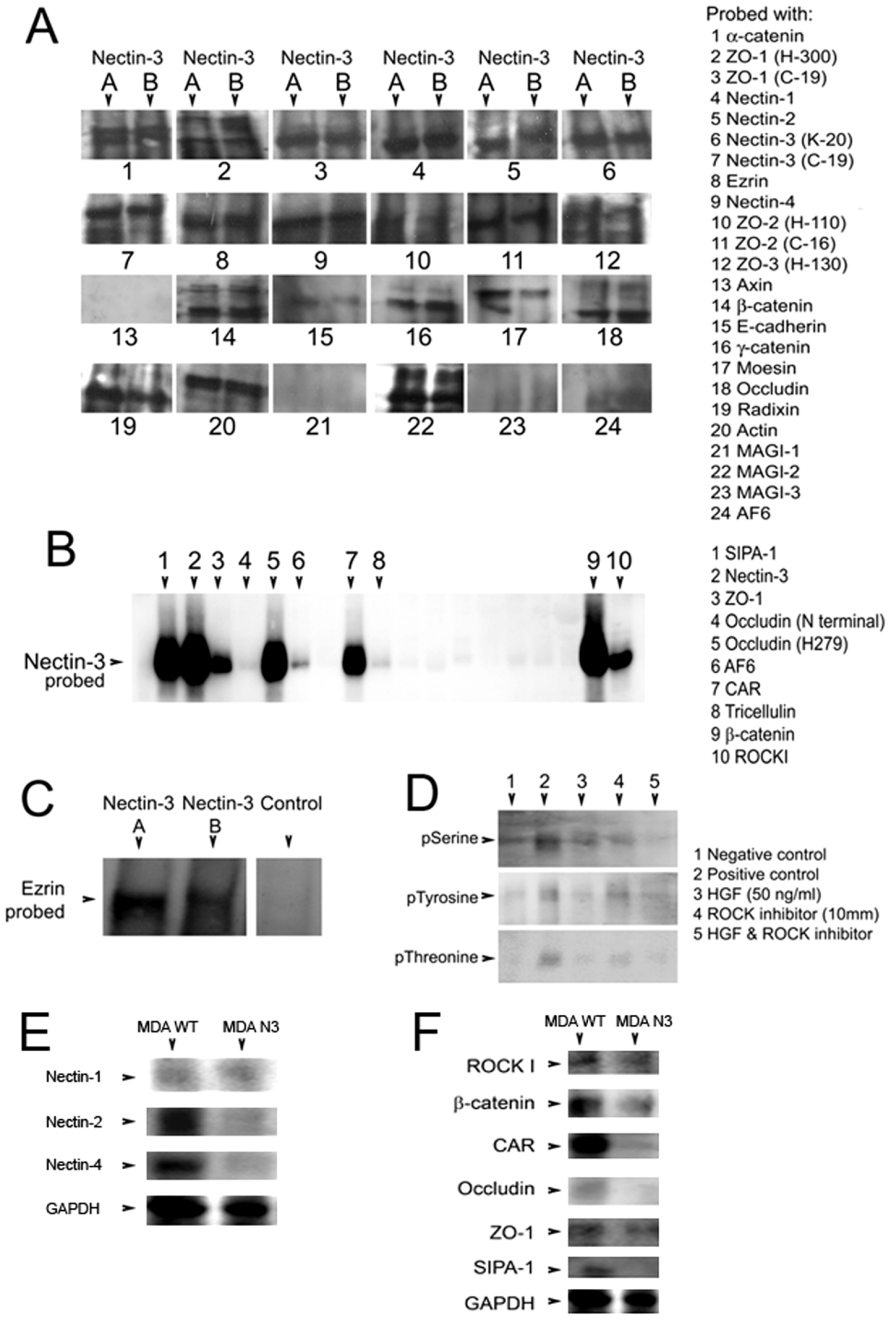
Immunoprecipitation study of Nectin-3 (A, an antibody to the C-terminal region; B, an antibody to an internal region) and proteins that are involved in cell to cell adhesion (A). Immunoprecipitation of relevant proteins probed with Nectin-3 (B). Ezrin precipitation and confirmation of Nectin-3 interaction (C). Phosphorylation study of Nectin-3 after treatment with HGF (40 ng/ml) and/or the ROCK inhibitor Y-27632 (D). Effect of Nectin-3 over-expression on the protein expression of Nectin-1, -2 and -4 (E). Effect of Nectin-3 over-expression on the protein expression of potential binding partners (F).

Due to the discrepancy in expression of Nectin-3, as seen from RT-PCR, we decided to investigate the binding of Nectin-3 in cells using two antibodies, (A) which binds to the C-terminus of Nectin-3 and (B) which binds to an internal region. A number of immunoprecipitates were then carried out with both antibodies, in order to determine possible binding partners of Nectin-3. Figure 6A shows the Immunoprecipitation results of this. There was, overall, little difference in precipitations between the two different antibodies used, (A) mapping to the C-terminus of Nectin-3, (B) mapping to an internal region. Positive precipitations were observed for α-catenin, ZO-1 (at both the C-teminus and an internal antibody), Nectin-1 and -2, Ezrin, Nectin-4, weakly for ZO-2 at the internal region, but strongly at the C-teminus, weakly for ZO-3. There was also interaction to β-catenin, γ-catenin and Moesin (two isoforms showing for (A), weak signal for (B)), Radixin and Actin. There was also a strong precipitation with MAGI-2. We also repeated the precipitation with a selection of the antibodies and probed with Nectin-3 (A) and found strong precipitation with SIPA-1, ZO-1, Occludin (at an internal region), CAR, β-catenin and ROCKI (Figure 6B). We were surprised to see the precipitation with Ezrin and again repeated this precipitation with both (A) and (B)- again, this was positive (Figure 6C).

### The phosphorylation status of Nectin-3, the effect of HGF and ROCK inhibitor

As over-expression of Nectin-3 in both MDA-MB-231 and HECV cells prevented the effect of HGF on cell motility, barrier function and invasion or tubule formation (all key contributors to metastasis) we went on to determine if HGF exerted any effect on the phosphorylation status of the protein. From the Immunoprecipitation shown in Figure 6D, it appeared that HGF exerted little effect on serine phosphorylation of Nectin-3, and no effect on either tyrosine or threonine phosphorylation. As we were surprised to observe an interaction between Nectin-3 and ROCKI, we looked at the effect of the ROCK inhibitor (y-27632) on Nectin-3 phosphorylation. We found an increased tyrosine phosphorylation of Nectin-3 after ROCKI inhibitor (10 mm) treatment over 30 mins (Figure 5D, middle). There was also a weak increase in threonine phosphorylation (Figure 5D, bottom). Western blotting was then carried out to determine any changes in expression of the other Nectin proteins after Nectin-3 over-expression in MDA-MB-231 cells (Figure 6 E). It can be seen whilst there was no difference in protein levels for Nectin-1, both Nectin-2 and Nectin-4 showed some reduction in levels. We then went on to investigate if Nectin-3 over-expression caused a change in protein levels of the proteins we found to have potential binding interactions with Nectin-3 (Figure 6 F). Surprisingly, the over-expression of Nectin-3 in these cells resulted in decreased protein expression of β-catenin, CAR and SIPA-1. There was no difference in expression of ROCK I and ZO-1.

## Discussion

The Nectin protein family is still little investigated in cancer. Our study has shown that high levels of Nectin-1 and Nectin-2 is associated with poor prognosis and patient outcome in human breast cancer. Previous studies on Nectin-1 have concentrated on its role in sensitivity to herpes oncolytic therapy in squamous cell carcinoma, thyroid cancer and head/neck carcinoma [20]–[21]. However, it has been demonstrated that there is a link between reduction in breast cancer cell invasion caused by SNAI1-triggered epithelial to mesenchymal transition (EMT) and the down-regulation of Nectin-1 [22]. The over-expression of Nectin-2 has previously been described in breast and ovarian cancer tissues using gene arrays and immunohistochemistry [13]. The authors further determined that Nectin-2 was over-expressed in various breast and ovarian cell lines using flow cytometry, concluding that Nectin-2 could serve as a target for antibody therapy in these cancer types. Increased levels of Nectin-2 have also been found to be a biomarker for poor prognosis and metastatic disease in squamous cell and adenosquamous carcinoma and adenocarcinoma of the gallbladder [23].

We found Nectin-3 and Nectin-4 to be reduced in breast cancer and associated with good prognosis and patient outcome. However, in lung adenocarcinoma, it has been reported that membranous expression of Nectin-3 is an independent prognostic indicator [17]. Interestingly, the authors demonstrated this was only true in patients where Nectin-3 did not co-localise with E-cadherin; where there was co-localisation, patient prognosis was favourable. In contrast to the results described here, an earlier study on Nectin-4 using a smaller cohort, described no expression in normal breast epithelium, but expression in 61% of the ductal cancers examined and that nearly all ER/PR negative tumours expressed Nectin-4 [16]. When testing serum from patients, the authors found there to be a correlation between serum Nectin-4 and disease progression. In ovarian cancer, Nectin-4 has also been reported to be increased in tumour cells and tissues, compared to normal [15].

Our immunohistochemical staining for Nectin-3 in human breast tissues revealed the protein to be concentrated as small inclusions in the nuclear region. These inclusions were seen only in cells from tumour tissues. This could have a direct bearing on the role of Nectin-3 in the cell. Nectin-3 was first described by Satoh-Horikawa et al. [6]. They found a novel Ca2+independent homophilic binding cell-cell unit located at cadherin based adherens junctions. The authors isolated three splicing variants which were nectin-3α (largest), -3β (middle), and -3γ (smallest). Nectin-3α was found to consist of three extracellular domains, a transmembrane region and a cytoplasmic tail with a PDZ-binding motif. Nectin-3α formed a *cis*-homo-dimer and showed Ca2+-independent *trans*-homo-interaction to cause homophilic cell-cell adhesion. Nectin-3α furthermore showed *trans*-hetero-interaction with nectin-1 or -2 but did not form a *cis*-hetero-dimer with nectin-1 or -2. Moreover, Nectin-3α interacted with actin and colocalised with Nectin-2 [6]. In comparison, nectin-3γ lacked the C-teminal PDZ motif and was unable to interact with actin. In our current study, all four Nectins had aberrant expression in the cancer cells lines investigated. Nectin-3 was only fully expressed in one breast cancer cell line (BT-482). Sequential overlapping amplification via RT-PCR showed that the majority of breast cancer cell lines expressed the first domain of Nectin-3. This truncated Nectin-3 does not correspond to any of the Nectin-3 spliced variants, being too short. Interestingly, increased confluency of human breast cancer cells resulted in increased transcript of Nectin-3.

Over-expression of Nectin-3 in human breast cancer cells resulted in a significantly reduced aggressive phenotype, cells that were less motile, less invasive, slower growing; however, these cells had increase TJ function. In addition, expression of Nectin-3 in human endothelial cells also imbued cells with increased barrier function and reduced ability to undergo tubulogenesis. It appears that Nectin-3 could be involved in barrier function and that the aberrant expression observed in wild type cells could prevent correct assembly of cell-cell junctions. Each member of the Nectin family forms homo-cis-dimers, followed by formation of homo-trans-dimers, causing cell-cell adhesion [24]. Nectin-3 also forms hetero-trans-dimers with either Nectin-1 or Nectin2 and the formation of these is much stronger than that of the homo-trans-dimers. It has been shown that the first Ig-like domain of Nectins-1, -2, and -3 contain a highly conserved peptide sequence that corresponds to aa 118–132, aa 125–139, and aa 142–156, respectively (6). The authors drew an analogy with E-cadherin, where this conserved sequence is crucial for trans-interaction between cells. Nectin-3α does not form a *cis*-hetero-dimer with Nectin-1α or -2α and it is likely that a portion(s) of the extracellular region, which is different from that necessary for *trans*-interaction, determines *cis*-dimerization specificity [6]. Nectins have the potential to recruit the E-cadherin-beta-catenin complex to the Nectin based cell-cell adhesion sites through afadin and α-catenin [25]. Moreover, Nectins have been shown to recruit ZO-1 also [26]. Fukuhara et al. [27] have shown that Nectin-1 plays a role in the localisation of TJ components, Claudin-1 and occludin, in the formation of the junctional process in MDCK cells. Claudin-1 and occludin accumulated at the apical sites of Nectin-1α-based cell-cell adhesion sites during the formation of the junctional complex. The accumulation of Claudin-1 and occludin could be inhibited by Nectin inhibitors, gD and Nef-3, which inhibited the trans interaction of Nectin-1α. Nectin inhibitors also impaired the barrier function of TJ [27]. Such results suggest that trans interaction of Nectin-1 is necessary for the localisation of Claudin-1 and occludin as well as the formation of TJs. Claudin-1 and occludin interact with ZO-1 through its C-terminal and Nectin-1 recruits ZO-1 to the Nectin based cell-cell adhesion sites through afadin in a cadherin-independent manner [26]. Thus Nectin-1 recruits Claudin-1 and occludin through their cytoplasmic-tail binding proteins afadin and ZO-1. Nectin-1 is also involved in the localisation of JAM at TJs [27]. During the formation of cell-cell junctions, the trans-interaction of Nectins first occurs at the initial cell-cell contact sites, and then promotes the formation of cadherin-based AJs and the subsequent formation of claudin-based TJs [28]. It can therefore be postulated that Nectin-3 has a hitherto unreported role in the successful organization of TJs. The Immunoprecipitation experiments we carried out showed a number of potential binding partners amongst well known TJ proteins that could suggest a similar role for Nectin-3 in the recruitment of TJ components to the cell membrane. This could be an essential mechanism in breast epithelial cells to maintaining cell adhesion and barrier function. In cancer cells therefore, aberrant expression of Nectin-3 could lead to the prevention of TJ protein recruitment, lack of formation of TJs and hence loss of cell-cell integrity. Increasing evidence has placed TJs as the key structure that cancer cells must overcome in order to successfully metastasize [29].

Also of interest was the possible interaction of Nectin-3 with proteins from the ERM family, i.e. ezrin, radixin and moesin. The ERM protein family act as molecular cross-linkers between actin filaments and proteins anchored in the cell membrane [30]. They participate in a complex intracellular network of signal transduction pathways and play a key role in the regulation of adhesion and polarity of normal cells through interactions with various membrane molecules. Ezrin and related molecules are concentrated at surface projections such as microvilli and membrane ruffles where they link the microfilaments to the membrane. Actin binding proteins allow cross-linking of actin filaments and regulation of actin filaments prior to cell motility. ERM proteins are believed to act as membrane organisers and linkers between plasma membrane molecules such as CD44 and ICAM-2 and the cytoskeleton [31]–[32]. There is now compelling scientific and clinical evidence that adhesion molecules and the ezrin family are important structures in controlling cell functions such as adhesion as well as controlling the progressive nature of cancer cells [33]. It is therefore probable that control of the assembly or disassembly of cell-cell adhesion and changes in the cell actin cytoskeleton leading to motility could involve some interaction between Nectin-3 and ezrin, for example. This is an area that could be fertile for further research.

The possible interaction between ROCKI and Nectin-3 could also be another exciting area of research. Changes in the phosphorylation status of Nectin-3 shown in this current study, demonstrates that the Y-27632 ROCK inhibitor increased both tyrosine and threonine phosphorylation. There have been no studies investigating the phosphorylation status of Nectin-3 and limited studies on the phosphorylation of other Nectins. Nectin-2δ is tyrosine phosphorylated in response to cell-cell adhesion [8] and knockdown of afadin or Nectin-3 in NIH3T3 cells caused relatively rapid suppression of the PDGF (platelet-derived growth factor)-induced phosphorylation of Akt; moreover, an increase in the phosphorylation of Akt occurred in afadin- or Nectin-3-knockdown NIH3T3 cells after treatment with PDGF [34], suggesting that the Nectin-afadin complex is involved in the (PDGF)-induced activation of phosphatidylinositol 3-kinase (PI3K)-Akt signaling for cell survival.

## Conclusion

In conclusion, it appears that Nectin family members have disparate expression in human breast cancer and that the aberrant expression of Nectin-3 is associated with metastatic disease. The expression and interaction of Nectin-3 in breast cancer and endothelial cells indicates that Nectin-3 may be a key component in the formation of cell junctions and be a putative suppressor molecule to the invasion of breast cancer cells.
